# *Mammalian orthoreovirus* Infection is Enhanced in Cells Pre-Treated with Sodium Arsenite

**DOI:** 10.3390/v11060563

**Published:** 2019-06-18

**Authors:** Michael M. Lutz, Megan P. Worth, Meleana M. Hinchman, John S.L. Parker, Emily D. Ledgerwood

**Affiliations:** 1 Department of Biological and Environmental Sciences, Le Moyne College, Syracuse, NY 13214 USA; Michael_Lutz@urmc.rochester.edu (M.M.L.IV); worthmp@lemoyne.edu (M.P.W.); 2 J.A. Baker Institute for Animal Health, College of Veterinary Medicine, Cornell University, Ithaca, NY 14853, USA; mmh46@cornell.edu (M.M.H.); jsp7@cornell.edu (J.S.L.P.)

**Keywords:** reovirus, viral factory, integrated stress response, translation, phosphorylated eIF2α, stress granules, HRI kinase, sodium arsenite, heat shock, salubrinal

## Abstract

Following reovirus infection, cells activate stress responses that repress canonical translation as a mechanism to limit progeny virion production. Work by others suggests that these stress responses, which are part of the integrated stress response (ISR), may benefit rather than repress reovirus replication. Here, we report that compared to untreated cells, treating cells with sodium arsenite (SA) to activate the ISR prior to infection enhanced viral protein expression, percent infectivity, and viral titer. SA-mediated enhancement was not strain-specific, but was cell-type specific. While SA pre-treatment of cells offered the greatest enhancement, treatment within the first 4 h of infection increased the percent of cells infected. SA activates the heme-regulated eIF2α (HRI) kinase, which phosphorylates eukaryotic translation initiation factor 2 alpha (eIF2α) to induce stress granule (SG) formation. Heat shock (HS), another activator of HRI, also induced eIF2α phosphorylation and SGs in cells. However, HS had no effect on percent infectivity or viral yield but did enhance viral protein expression. These data suggest that SA pre-treatment perturbs the cell in a way that is beneficial for reovirus and that this enhancement is independent of SG induction. Understanding how to manipulate the cellular stress responses during infection to enhance replication could help to maximize the oncolytic potential of reovirus.

## 1. Introduction

Acute viral infection induces stress within infected cells. The integrated stress response (ISR) is activated in many cells during viral infection [1,2,3]. The ISR facilitates cellular survival and a return to homeostasis, or initiates cell death signaling under conditions of severe stress or when the initiating stressor is maintained [4]. Four distinct stress kinases can be activated in response to stress. Although these kinases may have a number of substrates, they all phosphorylate the alpha subunit of the eukaryotic translation initiation factor 2 (eIF2α) at serine position 51. When eIF2α is phosphorylated, the GTP-exchange factor, eIF2B is unable to exchange GDP for GTP on eIF2. As a consequence eIF2B becomes sequestered and its effective levels drop, preventing efficient formation of the eIF2.GTP.Met-tRNAi ternary complex and thus inhibiting translation initiation [4]. 

The four cellular kinases known to phosphorylate eIF2α are heme-regulated eIF2α kinase (HRI), general control non-depressible 2 (GCN2), double-stranded RNA-dependent protein kinase (PKR), and PKR-like ER kinase (PERK) [4]. Both cell intrinsic and extrinsic stresses can activate these kinases: (i) HRI—heme-deprivation and oxidative stress; (ii) GCN2—amino acid starvation; (iii) PKR—accumulation of double-stranded RNA (as can occur during viral infection); and (iv) PERK—accumulation of unfolded proteins in the endoplasmic reticulum (ER) [4]. However, redundancy does exist amongst the kinases. For example, GCN2 is activated in response to ER stress in cells lacking PERK, and all four kinases can be activated under oxidative stress [5,6,7,8,9]. 

Following kinase phosphorylation of eIF2α, translation initiation is reduced and de novo protein synthesis is inhibited [4]. In response to this, stress granules (SGs) form in the cytoplasm. SG assembly is mediated by the RNA binding proteins, T-cell antigen 1 (TIA-1), TIA-1 related protein (TIAR), and the Ras-GAP SH3-binding protein 1 (G3BP1), and results in the compartmentalization of translationally-stalled mRNA transcripts, RNA-binding proteins, 40S ribosomes, and translation initiation factors [10,11]. SGs are considered to be sites of mRNA triage, protecting mRNA transcripts until a stress is alleviated and the cell returns to homeostasis.

Many viruses prevent activation of the ISR to maintain protein translation and to ensure successful viral infection. To do this, viruses target the initiating kinases and/or the downstream effector mechanisms of the ISR. Cells in which the initiating kinases, such as PKR, are inhibited or knocked out are often more permissive for viral replication [12,13]. However, not all viruses benefit from inhibition of kinase activity and initiation of the ISR. A study examining the role of the PKR-eIF2 pathway during *Mammalian orthoreovirus* (reovirus) infection found some strains had reduced titers in PKR knockout murine embryonic fibroblasts (MEFs) [14]. Follow-up studies observed both increased ISR gene expression and reduced levels of the eIF2α kinase inhibitor, P58^IPK^, in cells infected with reovirus strains known to robustly interfere with host translation, and these strains replicated less efficiently in MEFs expressing a non-phosphorylatable form of eIF2α [15]. 

Reovirus infection also modulates SG formation that occurs downstream of ISR activation [15,16]. Early in infection, entering viral core particles localize to SGs that form within infected cells. However, within 4 to 6 h after infection, the SGs have disappeared and viral factories (VFs), the sites of reovirus replication, translation, and assembly, become prominent [16,17,18,19]. In some reovirus-infected cells, the SG protein, GTPase-activating protein (SH3 domain) binding protein 1 (G3BP1), localizes to the margins of the VFs, mediated by an interaction of G3BP1 with the non-structural viral protein, σNS [20]. σNS also interacts with the nonstructural protein, μNS, that forms the matrix of VFs, and co-expression of σNS and μNS is sufficient to alter the localization of G3BP1 and suppress SG induction [20,21]. The interplay between eIF2α phosphorylation, PKR activation, translational shutoff, and G3BP1-induced SG formation is strain-dependent, as SG formation negatively impacts the replication of some strains of reovirus [20]. Together, these studies suggest a unique role for the ISR during reovirus infection. Given the previous observation that reovirus replicates to lower titers in cells with an impaired ISR, we hypothesized that reovirus infection would be enhanced in cells in which the ISR has been activated prior to infection. To test this, we utilized the chemical sodium arsenite (SA). When applied to cells, SA leads to the production of reactive oxygen species (ROS) and oxidative stress. In response to increased ROS levels, the HRI kinase phosphorylates eIF2α and induces SG formation, ultimately leading to translation inhibition [22]. We found that when we activated the ISR by pre-treating cells with SA prior to virus adsorption, reovirus infection was more efficient (increased permissivity, protein expression, and replication). SA-induced enhancement of reovirus infection was observed in all reovirus strains tested but was dependent on cell-type and the time of SA addition. Enhancement of viral infectivity was only observed if SA was added to cells within 4 h of inoculation, with maximal enhancement if the addition occurred prior to inoculation, suggesting a relationship between the ISR and early replication events. Furthermore, not all activators of the ISR were equally beneficial as heat shock (HS) prior to infection had no impact on viral replication. Taken together, these data suggest a critical role for the ISR during reovirus infection and that activation of the ISR with SA prior to reovirus infection is beneficial in some cell types. This study expands upon the previous observation that reovirus replicates to reduced titers in cells lacking a phosphorylatable eIF2α and helps to clarify uncertainty in the field as to the timing of SG induction by reovirus. Reovirus is an oncolytic virus, preferentially infecting and lysing cancer cells. Insight into how reovirus infection activates the stress response, and how these responses can be modulated to enhance infection, could help to maximize the oncolytic potential of this virus.

## 2. Materials and Methods

### 2.1. Cells and Reagents

CV-1 (CCL-70, ATCC, VA, USA) and HeLa cells (ATCC) were maintained in Eagle’s minimum essential medium (MEM) (CellGro; Corning, NY, USA)) containing 10% fetal bovine serum (FBS; Hyclone; GE Life Sciences, USA), 100 mM sodium pyruvate (CellGro), and 200 mM l-glutamine (CellGro) at 37 °C in the presence of 5% CO_2_. L929 cells were maintained in MEM containing 8% FBS and 200 mM L-glutamine at 37 °C in the presence of 5% CO_2_. Human pancreatic ductal epithelial (HPDE) cells (Kerafast H6c7; MA, USA) were maintained in keratinocyte SFM (Invitrogen, USA) supplemented with 25 mg bovine pituitary extract and 2.5 μg human recombinant epidermal growth factor, both provided with media, at 37 °C in the presence of 5% CO_2_. SA obtained from Sigma-Aldrich was kindly donated by Dr. Shu-bing Qian (Cornell University) and was used at a final concentration of 0.5 mM in all experiments.

### 2.2. Viruses

Reoviruses, Type 1 Lang (T1L) and Type 3 Dearing (T3D), laboratory stocks originated from the T1/human/Ohio/Lang/1952 and T3/human/Ohio/Dearing/1955 isolates respectively and represent clonal isolates from the M.L. Nibert laboratory (Harvard Medical School) [23,24]. The prototype reovirus serotype 3 strain Abney (T3A) was a kind gift from Dr. Barbary Sherry (North Carolina State University). Each virus was twice plaque-purified and passed two or three times on L929 cell monolayers before being subjected to three freeze–thaw cycles. Working L929 cell lysate virus stocks were titered on L cells, or by immunofluorescence staining for μNS as noted in the text, before use in experiments.

### 2.3. Infections

CV-1, L929, HeLa, or HPDE cells were seeded in 24-well culture plates containing 12 mm glass coverslips or 12-well culture plates the day before to give rise to 50% to 80% confluence prior to infection. Cells were infected with virus at the indicated multiplicity of infection (MOI) for 1 h at room temperature (RT) in phosphate-buffered saline (PBS; pH 7.4), supplemented with 2 mM MgCl_2_, with rocking every 10 min. Following absorption, virus was removed and cells were incubated in growth medium at 37 °C and harvested at the indicated time points. 

### 2.4. SG Induction

SGs were induced in cells using the following mechanisms: 1) Treatment with 0.5 mM SA in normal growth media at 37 °C for 30 min prior to infection or 30 min prior to harvesting, or 2) HS in normal growth media in a pre-heated incubator at 44 °C for 45 min prior to infection or harvesting. Cells were manually determined to contain SGs if immunofluorescence revealed a minimum of three granules co-staining for both TIAR and G3BP.

### 2.5. Antibodies

The following commercial primary antibodies were used for immunoblotting: Mouse monoclonal anti-G3BP1 (2F3) antibody (H00010146-M01; Novus Biologicals, CO, USA), mouse monoclonal anti-TIAR antibody (sc-398372; Santa Cruz Biotechnology, TX, USA), mouse monoclonal anti-eIF2α (sc-133132; Santa Cruz Biotechnology), rabbit polyclonal anti-peIF2α S51 (GTX50300; GeneTex, CA, USA) mouse monoclonal anti-α-tubulin antibody (NB100-690; Novus Biologicals), and mouse monoclonal anti-HRI (sc-365239; Santa Cruz Biotechnology). Reovirus protein expression was assessed using a chicken polyclonal antiserum against μNS prepared against bacterially-expressed purified antigen (Covance) or mouse monoclonal anti-μ1 (clone 4A3), a kind gift from Dr. Terence Dermody (University of Pittsburgh) [25]. Secondary antibodies used for immunoblotting were as follows: HRP Donkey Anti-Mouse IgG (715-035-150; Jackson ImmunoResearch, PA, USA), and HRP Donkey Anti-Chicken IgY (703-035-155; Jackson ImmunoResearch). Primary antibodies used for immunofluorescence included: Mouse monoclonal anti-G3BP1 (611126; BD Biosciences, CA, USA), rabbit monoclonal anti-TIAR (8509S; Cell Signaling Technology, MA, USA), and anti-μNS to detect viral factories. Secondary antibodies used for immunofluorescence assays included Alexa Fluor 594 goat anti-chicken IgG, Alexa Fluor 594 goat anti-rabbit IgG, Alexa Fluor 488 goat anti-mouse IgG, and Alexa Fluor 488 goat anti-rabbit IgG (Thermofisher, NY, USA).

### 2.6. Immunofluorescence

Cells were washed once with PBS supplemented with 2 mM MgCl_2_ and fixed at room temperature for 10 min with 4% paraformaldehyde in PBS. Fixed cells were washed once with PBS, permeabilized in 0.1% Triton X-100 in PBS for 15 min, and washed three times with PBS. Cells were blocked for 15 min in staining buffer (SB; 0.05% saponin, 10 mM glycine, 5% FBS, and PBS) and incubated with primary antibodies diluted in SB for 1 h. Cells were then washed one time with PBS before incubation with secondary antibodies diluted in SB for 1 h. Coverslips were mounted onto glass slides with ProLong Gold Anti-Fade reagent with DAPI (4[prime],6-diamidino-2-phenylindole; ThermoFisher). Images were obtained using an Olympus BX60 inverted microscope equipped with phase and fluorescence optics. Images were collected digitally with an Olympus DP74 color CMOS camera and cellSens Standard Software (Olympus, USA) and were processed and prepared for presentation using Photoshop (CC; Adobe, CA, USA) software. To determine the percent cells containing SG or VF, a minimum of three individual experiments was performed. Within each experiment, a minimum of four fields of view were manually counted totaling no less than 100 cells per condition. All fields were selected randomly by scanning exclusively under the dapi channel. Cells were considered to be positive for SG if they contained a minimum of three granules co-staining for TIAR and G3BP. Cells were considered to be infected if they contained μNS inclusions (VF).

### 2.7. Immunoblot Assay

Cells were lysed in PBS containing 0.5% NP40, 140 mM NaCl, 30 mM Tris-HCl (pH 7.4), and an EDTA-free protease inhibitor cocktail (04693159001; Sigma Aldrich, MO, USA) for 30 min on ice before clarifying by centrifugation and retaining the supernatant. Cell lysates were resolved by SDS-PAGE and proteins were detected using antibodies as described above. Images were collected using a C-Digit digital scanner and Image Studio Digits software (Version 4; LiCor, NE, USA). When appropriate, immunoblots were incubated in stripping buffer (200 mM glycine, 3.5 mM SDS, 1% Tween 20, (pH 2.2)) and reprobed. Densitometry analysis was performed by measuring the intensity of each band using Image Studio Digits software. Relative density = normalized density target protein/normalized density loading control.

### 2.8. Plaque Assay

Cells were infected as above before washing with PBS supplemented with 2 mM MgCl_2_ and incubated in growth medium at 37 °C for the indicated times. Cells were subjected to two freeze/thaw cycles prior to the determination of viral titer by the standard reovirus plaque assay in L929 cells [26]. Briefly, 10-fold serial dilutions of lysates were used to infect monolayers of L929 cells before overlaying with agar containing 10 μg/mL TLCK α-chymotrypsin (Sigma Aldrich, MO, USA) to facilitate plaque formation. Following incubation at 37 °C for 3 days, plaques were visualized and counted. To determine the viral titer in plaque-forming units (PFU)/mL, the following equation was used: PFU/mL = number of plaques/(D × V), where D = dilution factor and V = volume of diluted virus/well.

## 3. Results

### 3.1. Infection with Reovirus Induces SG Formation

Previous reports have suggested that SGs form following infection with reovirus, but reports are conflicting as to when SGs appear and when they disappear in infected cells [15,16]. We selected to perform our analyses in CV-1 African green monkey kidney cells, a commonly used line in the reovirus field. First, we confirmed that SGs could form in CV-1 cells by treating uninfected cells with 0.5 mM SA for 30 min. 

**Figure 1:**
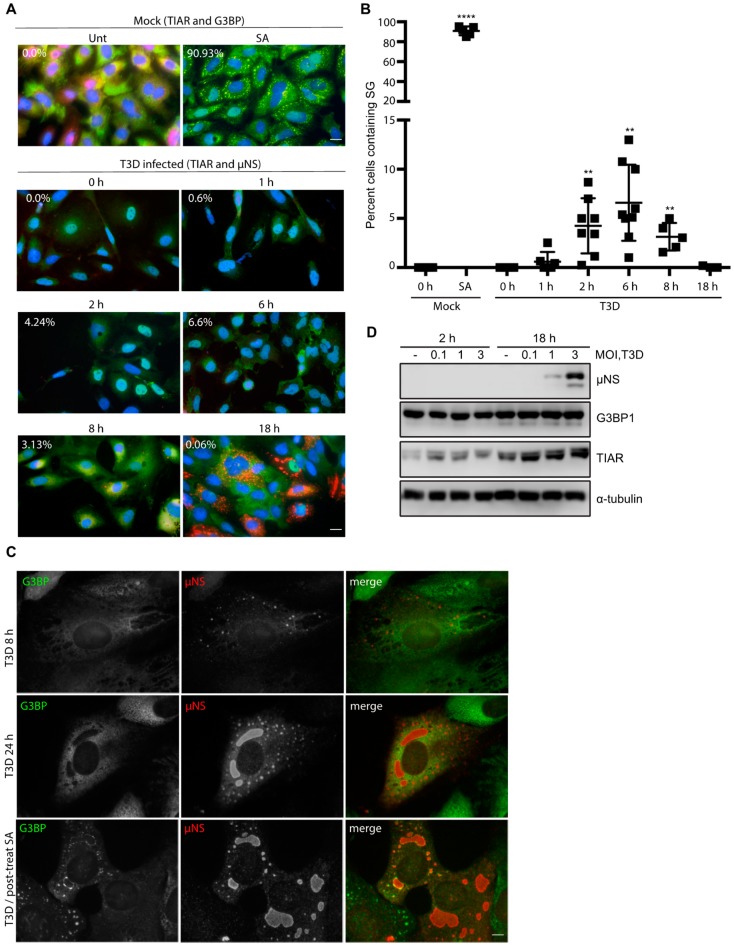
Reovirus infection induces the formation of stress granules (SGs) at early times post infection (**A**) CV-1 cells were mock infected, pre-treated with 0.5 mM sodium arsenite (SA) for 30 min, or infected with type 3 Dearing (T3D) at a multiplicity of infection (MOI) = 10. At the indicated times, cells were fixed and co-immunostained for G3BP (red) and TIAR (green), upper panels, to detect SGs in uninfected cells or TIAR (green) and μNS (red), lower panels, to detect SGs in T3D-infected cells. Cell nuclei were stained with DAPI (blue). Mock cells were fixed at 18 h. Scale bars, 20 μm. (**B**) The percent of cells containing SGs from panel (A) was quantified ((# of cells containing SGs/total # of cells) × 100) from a minimum of three independent experiments. ** *p* < 0.01, **** *p* < 0.0001; two-tailed unpaired t test. The error bars indicate S.D. (**C**) CV-1 cells were infected with T3D at an MOI = 0.5 and at 8 and 24 h p.i. cells were fixed and co-immunostained with G3BP (green) and μNS (red), upper panels. Alternatively, CV-1 cells were infected with T3D at an MOI = 1 and at 23.5 h cells were post-treated with 0.5 mM SA for 30 min. At 24 h p.i., cells were fixed and co-immunostained with G3BP (green) and μNS (red), lower panels. Scale bars, 10 μm. (**D**) Cells were either mock infected (-) or were infected with T3D and, at the indicated times, cells were lysed and the expression level of proteins was determined by immunoblotting.

We then fixed and co-immunostained for TIAR and G3BP, two RNA binding proteins required for SG formation. We observed SGs in ~91% of the treated CV-1 cells, confirming that these cells can form SGs (Figure 1A, top row and Figure S1). We next evaluated the capacity of reovirus to induce SG formation in the absence of SA. To do this, we infected CV-1 cells with reovirus T3D at a multiplicity of infection (MOI) of 10 and, at the indicated times post-infection (p.i.), fixed and co-immunostained cells for the presence of SGs (TIAR) and VFs (μNS) (Figure 1A, lower panels and Figure S1). SGs were absent in mock-infected cells and began appearing in wells infected with T3D around 2 h p.i. We found that SG formation in reovirus-infected wells peaked around 6 h p.i. with 6.6% of total cells containing SGs. By 8 h p.i., the percentage of cells containing SGs had dropped to 3.1% and by 18 h p.i., the percentage of cells containing SGs was no different from mock-infected cells (Figure 1A,B and Figure S1). A previous report detected SGs in reovirus-infected human prostate carcinoma DU145 cells at 19.5 h post infection [15]. We were unable to detect SGs in reovirus infected cells at late times post infection at a variety of MOIs (Figure 1A (bottom row) and Figure 1C (middle row). However, G3BP did localize to the periphery of VFs in some infected cells at 24 h p.i. (Figure 1C, middle row). This was not seen at 8 h p.i. (Figure 1C, top row). Furthermore, if we treated infected cells with SA at 23.5 h p.i. immediately before harvest as others have done, G3BP could be observed in punctae at the periphery of VFs in some infected cells (Figure 1C) [15,20]. These findings are in agreement with those of Choudhury et al., who found that G3BP localizes to the VF periphery during reovirus infection [20]. To determine if reovirus altered the expression levels of SG proteins, we next evaluated G3BP and TIAR protein expression during infection (Figure 1D). While G3BP levels remained relatively constant over the course of infection, we did note an increase in TIAR expression at 18 h p.i. by immunoblot that was independent of MOI. Additionally, the increase in TIAR expression at 18 h was evident for both mock and reovirus-infected cells (Figure 1D). This finding is in contrast to the immunofluorescence data presented in Figure 1A, where no changes in overall TIAR expression were noted. However, we did observe that the localization of TIAR shifted over time, with TIAR expression primarily localized to the nucleus at early times post infection, but primarily found in the cytoplasm at late times post infection (Figure S1). Together, these data suggest that reovirus infection induces SGs at early, but not late times post-infection in CV-1 cells.

### 3.2. Pre-Treatment with 0.5 mM SA Enhances Reovirus Infectivity

**Figure 2:**
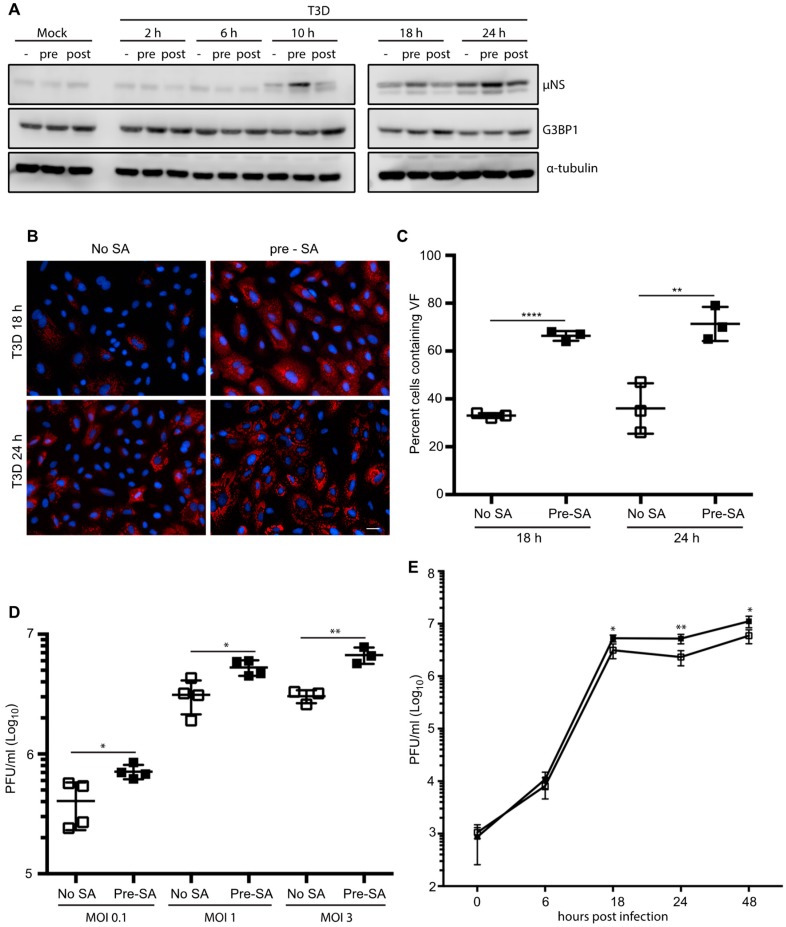
Pre-treatment with 0.5 mM SA enhances reovirus infectivity. (**A**) CV-1 cells were mock infected or infected with T3D at an MOI = 1. Cells were left untreated (-), treated with 0.5 mM SA for 30 min prior to infection (pre), or were treated with 0.5 mM SA for 30 min immediately before lysis (post). At the indicated time points, cells were harvested and protein expression was determined by immunoblot. (**B**) CV-1 cells were infected with T3D at an MOI = 1. Cells were left untreated (no SA) or were treated with 0.5 mM SA for 30 min prior to infection (pre-SA). At the indicated times, cells were fixed and immunostained for μNS (red) and DAPI (nuclei, blue). Scale bars, 50 μm. (**C**) The percent of cells containing viral factories (VFs) as represented in panel (B) was quantified ((# of cells containing VFs/total # of cells) × 100) from three independent experiments. (**D**) CV-1 cells were left untreated or were pre-treated with 0.5 mM SA for 30 min prior to infection with T3D at the indicated MOI. At 18 h p.i., titers of infectious virus in cell lysates were determined by plaque assay in L929 cells. Plaques were counted from at least three independent experiments. (**E**) CV-1 cells were left untreated (□) or were pre-treated with 0.5 mM SA (■) for 30 min prior to infection with T3D at an MOI = 1. At the indicated times p.i., titers of infectious virus in cell lysates were determined by plaque assay in L929 cells. Plaques were counted from at least three independent experiments. * *p* < 0.05, ** *p* < 0.01, **** *p* < 0.0001; two-tailed unpaired *t* test. The error bars indicate S.D.

Following infection with reovirus, the viral protein, μNS, orchestrates the formation of VFs, the sites of virus replication, assembly, and translation [17,18,19]. Cellular factors involved in translation, such as eIF3, eIF4G, and ribosomal subunits, are recruited to VFs and many of these initiation factors are similarly compartmentalized within SGs [19,27]. To date, most studies have focused on the induction of SGs in response to viral infection, or viral suppression of SG formation during an infection. To our knowledge, no studies have been performed to assess the impact of the presence of SGs on the initiation of reovirus infection. To explore this, we first examined the effect of SG presence on viral protein expression. CV-1 cells were infected with T3D at an MOI = 1 and were either pre-treated by adding 0.5 mM SA for 30 min before the start of infection (pre) or post-treated by adding SA for the 30 min immediately before harvest (post). Cell lysates were collected at 0, 2, 6, 10, 18, and 24 h p.i. and the expression level of the non-structural viral protein, μNS, was determined (μNS was first detectable by 10 h p.i.). At 10, 18, and 24 h p.i., the expression levels of μNS were consistently higher in cells pre-treated with SA when compared to untreated cells or cells treated with SA 30 min before harvest (Figure 2A). Consistent with Figure 1D, G3BP expression levels remained relatively constant throughout the course of infection, and in response to the various treatments (Figure 2A). 

As the viral protein, μNS, is necessary for the formation of VFs during reovirus infection, we next explored if the elevated μNS expression was a consequence of an increase in the number of infected cells [28]. We found that, in cells pre-treated with 0.5 mM SA for 30 min prior to reovirus infection, 67% of cells were infected (contained VFs) at 18 h p.i., whereas only 33% of untreated cells were infected at 18 h p.i. We observed a similar pattern at 24 h p.i. (Figure 2B,C).

Given the increased percentage of infected cells in dishes pre-treated with SA, we tested if the pre-treatment of cells also enhanced viral yield. We found that pre-treatment with SA led to a modest but significant 2- to 3-fold increase in viral titer (PFU/mL) that was independent of MOI, but was consistent with the increased numbers of infected cells and the increased protein expression (Figure 2D). To further assess the functional consequences of SA pre-treatment on reovirus replication, we performed a one-step growth curve in CV-1 cells infected with reovirus at an MOI = 1. While there was no difference between the two groups at early times following infection (6 h), the viral titer was consistently higher in cells pre-treated with SA at late times post infection (Figure 2E). Together, these data indicate that pre-treatment of cells with SA enhances reovirus infectivity by increasing the numbers of virus-permissive cells.

### 3.3. SA-Induced Enhancement of Reovirus Permissivity is Cell-Type Specific

**Figure 3:**
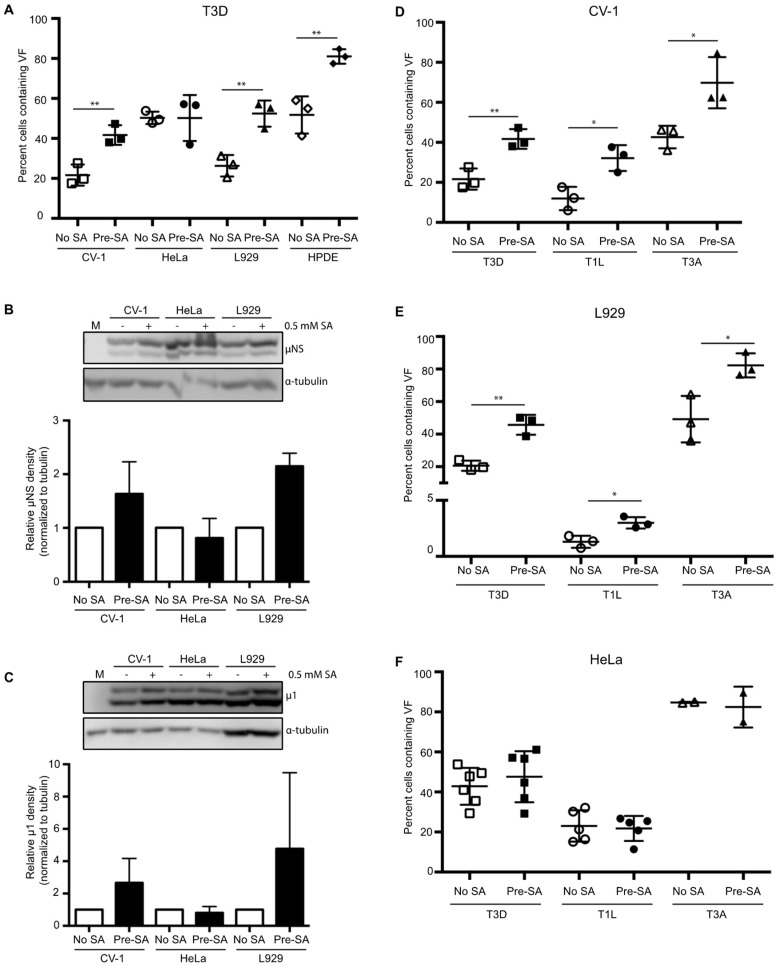
Pre-treatment with 0.5 mM sodium arsenite (SA) enhances permissivity in a cell-type-specific manner across reovirus strains. (**A**) CV-1, HeLa, L929, or HPDE cells were left untreated (no SA) or were treated with 0.5 mM SA for 30 min prior to infection (Pre-SA). Following this, cells were infected with T3D such that ~20% to 50% of cells were infected and at 18 h p.i. cells were fixed and immunostained for μNS and DAPI to visualize viral factories (VFs). The percent of cells containing VFs was quantified ((# of cells containing VFs/total # of cells) × 100) from three independent experiments. The expression level of μNS (**B**) and μ1 (**C**) was determined in CV-1, L929, or HeLa cells either left untreated (no SA) or treated with 0.5 mM SA for 30 min (Pre-SA) before infection with T3D at MOI = 1. At 18 h p.i., cells were harvested and the expression level of the indicated proteins was determined by immunoblot. M = mock. Densitometry analysis of the band intensity for μNS and μ1 was adjusted to the matched α-tubulin loading control for two independent experiments. Columns represent mean ± SEM. (**D**) CV-1; (**E**) L929; or (**F**) HeLa cells were left untreated (no SA) or were treated with 0.5 mM SA prior to infection (Pre-SA). Cells were then infected with the reovirus strains, T3D, T1L, or T3A, as described in (A). At 18 h p.i., cells were fixed and immunostained for μNS and DAPI to detect VFs. The percent of cells containing VFs was quantified ((# of cells containing VFs/total # of cells) × 100) from at least two independent experiments. * *p* < 0.05; ** *p* < 0.01; two-tailed unpaired *t* test. The error bars indicate S.D.

Previous reports have suggested that reovirus-induced SG formation is cell-type specific [15,16]. Therefore, we next sought to determine if the replication enhancement observed in CV-1 cells was seen in other cell types. To do this, we selected three additional cell types to represent a variety of species and tissue types to complement our studies performed in CV-1 African green monkey kidney cells. These included L929 murine fibroblasts, a cell line commonly used to grow reovirus; HeLa cells, an immortal human cervical epithelial cell line; and HPDE cells, a non-cancerous human pancreatic ductal epithelial cell line. We first evaluated whether SGs could form in each of these cell types. Treatment with 0.5 mM SA for 30 min induced strong SG formation in each line (Figure S2A). Next, we assessed the effect of pre-treatment with 0.5 mM SA on viral permissivity in each cell type. Since each cell line differed in its permissivity to reovirus, we performed immunofluorescence using antibodies against μNS to determine the concentration of virus required to give rise to 20% to 50% of the cells being infected. We chose 20% to 50% to ensure that any positive or negative effects of SA treatment were captured. Consistent with our observations in CV-1 cells, pre-treatment with SA in L929 and HPDE cells resulted in a higher percentage of cells exhibiting VFs than in untreated cells (Figure 3A). In HeLa cells, however, the percent of infected cells was comparable in both untreated and SA pre-treated cells (Figure 3A). To further assess the static permissivity in HeLa cells, viral protein expression was compared in HeLa, L929, and CV-1 cells. For this, we determined the expression levels of μNS and the reovirus outer capsid protein, μ1, in the presence or absence of SA pre-treatment [29]. Cells were pre-treated with SA for 30 min prior to infection with T3D at an MOI = 1, and at 18 h p.i., cells were harvested and the expression level of the indicated proteins was determined by immunoblot. Consistent with Figure 3A, at 18 h p.i., the expression level of μNS and μ1 was increased in CV-1 and L929 cells pre-treated with SA, but was unchanged in HeLa cells (Figure 3B,C). The difference observed between HeLa and CV-1 or L929 cells was not a result of a failure to induce SGs or phosphorylate eIF2α. Indeed, both of these outcomes occurred in HeLa cells in response to SA treatment (Figure S2A,B). These data indicate that activation of the stress response pathways prior to infection through pre-treatment with SA is beneficial in some cell types.

### 3.4. SA-Induced Enhancement of Reovirus Permissivity is Strain-Independent

The prototypic reovirus strains, Type 1 Lang (T1L), Type 3 Abney (T3A), and Type 3 Dearing (T3D), differ in their capacity to induce host translational shutoff and to induce eIF2α phosphorylation [15,30]. To determine if the benefit of pre-treatment with SA was reovirus strain specific, we infected CV-1 cells with T3D, T1L, or T3A and assessed the percentage of infected cells at 18 h p.i. compared to untreated cells. As observed following infection with the T3D strain, pre-treatment with SA resulted in nearly a 2-fold increase in the percentage of T1L- and T3A-infected CV-1 cells (Figure 3D). SA pre-treatment in L929 cells infected with T3D, T1L, or T3A yielded similar results, however, SA pre-treatment had no effect on any strain tested in HeLa cells (Figure 3E,F). These data suggest that the reovirus strains, T3D, T1L, and T3A, benefit from pre-treatment with SA prior to infection in some cell types.

### 3.5. HS Prior to Infection Enhances Viral Protein Expression in T3D-Infected L929 Cells, but Does not Affect the Percentage of Infected Cells or Viral Yield

**Figure 4:**
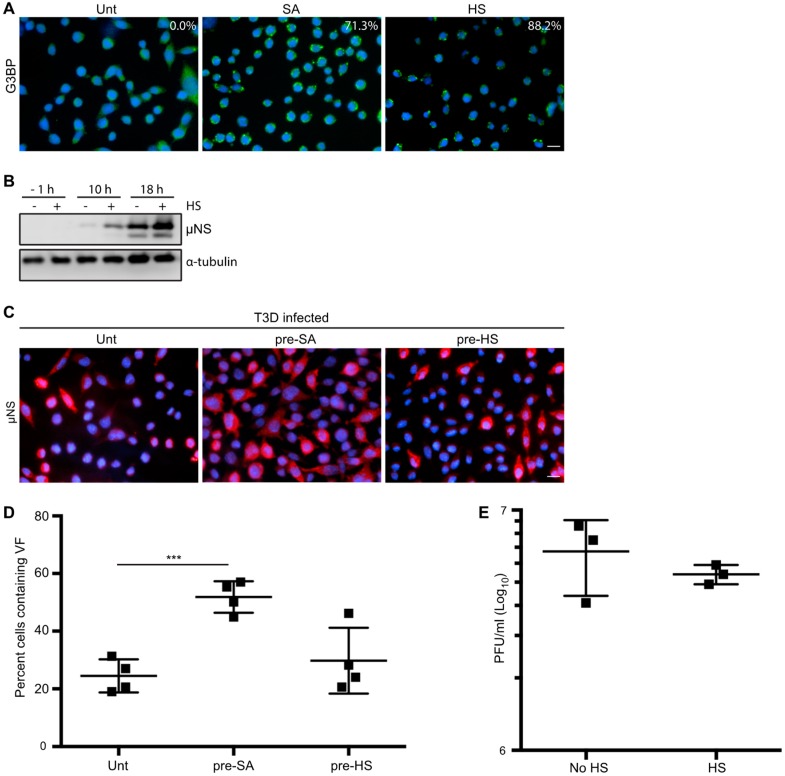
Heat shock (HS) enhances reovirus protein expression, but not virus yield, in L929 cells. (**A**) L929 cells were left untreated (Unt), were pre-treated with 0.5 mM sodium arsenite (SA), or were HS at 44 °C for 45 min (HS) before fixing and immunostaining for G3BP (green) to detect SGs. The percentage of SGs induced was quantified ((# of cells containing SGs/total # of cells) × 100) from three independent experiments. (**B**) L929 cells were left untreated or were HS as in (A) before infection with T3D at an MOI = 1. At the indicated time points, cells were lysed and the expression levels of the indicated proteins were determined by immunoblot. (**C**) L929 cells were left untreated, treated with 0.5 mM SA for 30 min, or were HS as in (A) before infection with T3D at an MOI = 1. At 18 h p.i., cells were fixed and immunostained for μNS (red) and DAPI (nuclei, blue). (**D**) The percent cells containing VFs as represented in panel (C) was quantified ((# of cells containing VF/total # of cells) × 100) from at least three independent experiments. (**E**) L929 cells were left untreated or were HS as in (A) prior to being infected with T3D at an MOI = 1. At 18 h p.i., cells were subjected to the standard reovirus plaque assay in L929 cells and plaques were counted from three independent experiments. *** *p* < 0.001; two-tailed unpaired *t* test. The error bars indicate S.D. Scale bars, 50 μm.

Cellular treatment with SA produces reactive oxygen species (ROS), which can accumulate, resulting in oxidative stress and activation of the HRI kinase [7,22,31]. The HRI kinase is activated in erythrocytes by reduced heme availability, but it is ubiquitously expressed in many tissues and, when activated by SA, phosphorylates eIF2α and subsequently induces SG formation [22]. HRI is the only stress kinase required for translational inhibition in response to arsenite treatment in mouse embryonic fibroblasts [22]. HRI kinase can also be activated by other cellular stresses, including osmotic stress and heat shock (HS) [7]. To further assess if the enhanced reovirus infectivity following SA treatment was specific to SA, we assessed viral protein expression, the percentage of infected cells, and the viral yield in response to HS. We used SG induction in response to HS as an indirect measure of HRI activation as we were unable to detect kinase expression by immunoblot using a commercial antibody (Figure 4A). Despite many attempts, we were unsuccessful at inducing SGs in more than 25% of CV-1 cells with HS. We also noted that HS failed to induce phosphorylation of eIF2α above levels found in untreated CV-1 cells (Figure S2B). However, we were readily able to induce SGs in L929 cells using HS. HS for 45 min at 44 °C resulted in ~88% of L929 cells containing SGs and an increase in eIF2α phosphorylation compared to untreated cells (Figure 4A and Figure S2B). Because of this, we assessed the effects of HS induction of the ISR in L929 cells. Similar to our observations in CV-1 cells pre-treated with SA, viral protein expression was enhanced at 10 and 18 h p.i. in L929 cells exposed to a 45 min HS prior to infection (Figure 4B). However, HS had no impact on the percentage of infected cells or the viral yield in L929 cells (Figure 4C–E). These findings suggest that in L929 cells, HS enhances protein expression but does not affect either the numbers of infected cells or the per cell yield of the virus. Although HS has previously been shown to activate the HRI kinase, its effect on cells differs from SA [7]. Together, these data indicate that SA pre-treatment perturbs the cell in a way that is beneficial for reovirus compared to other activators of the ISR.

### 3.6. Addition of SA Prior to 4 h Enhances Reovirus Permissivity

Activation of the ISR and eIF2α phosphorylation results in a suppression of general host translation [22]. While different reovirus strains induce translational shutoff to varying degrees, viral protein synthesis typically peaks around 12 h p.i. [32,33]. We found that viral protein expression was increased in CV-1 and L929 cells pre-treated with SA prior to infection and that the increase was absent if cells were treated immediately prior to harvest at 10, 18, or 24 h p.i. (Figure 2A and Figure 3B). Furthermore, viral protein expression, but not the percentage of infected cells or viral yield, was enhanced by activation of the ISR using HS in L929 cells (Figure 4). Given the impact on protein synthesis in multiple cell types following treatment with different stresses, we hypothesized that SA treatment might enhance viral translation at early times post infection. 

**Figure 5:**
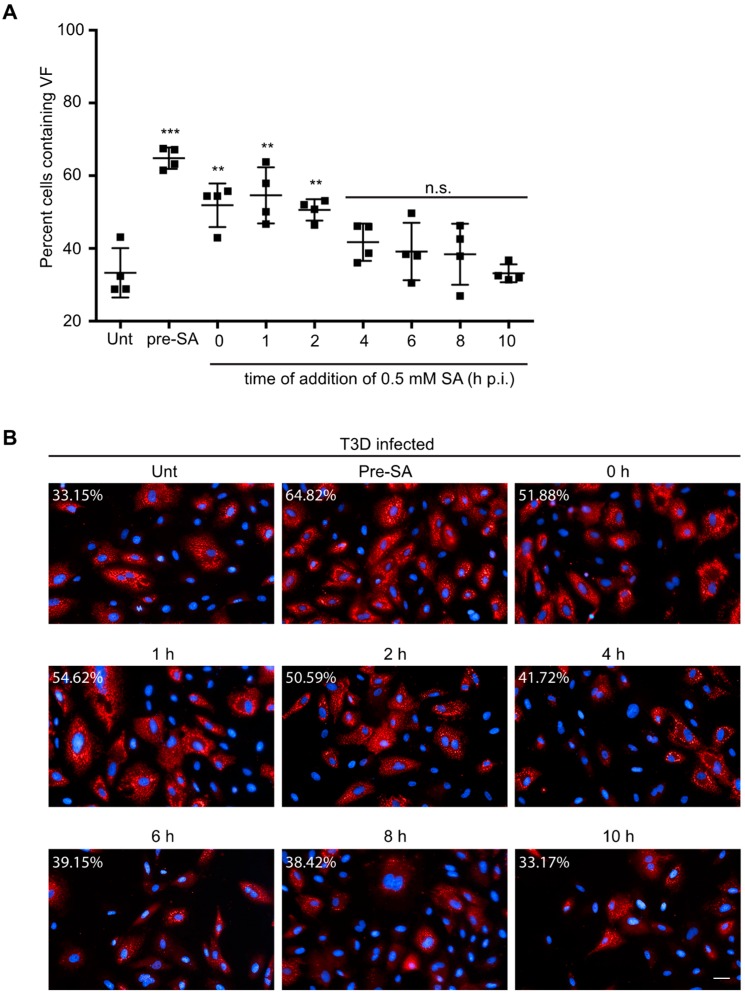
Addition of SA prior to 4 h p.i. enhances reovirus permissivity. (**A**) CV-1 cells were left untreated (unt), were pre-treated with 0.5 mM SA for 30 min prior to virus inoculation (pre-SA), or were treated with 0.5 mM SA for 30 min at the indicated times post infection. At 18 h p.i., cells were fixed and immunostained for μNS (red) and DAPI (nuclei, blue). (**B**) Representative images from each condition in (A). The percent of cells containing viral factories (VFs) was quantified ((# of cells containing VFs/total # of cells) × 100) from four independent experiments. ** *p* < 0.01; *** *p* < 0.001; n.s. denotes not significant; two-tailed unpaired *t* test. The error bars indicate S.D. Scale bars, 50 μm.

To test this, CV-1 cells were either left untreated (unt) or were pre-treated with 0.5 mM SA for 30 min prior to infection (pre-SA). Alternatively, following infection, 0.5 mM SA was added for 30 min at 0, 1, 2, 4, 6, 8, or 10 h p.i. We then assessed the percentage of infected cells in all treatment groups at 18 h p.i. Consistent with our previous data, pre-treatment with SA prior to inoculation with virus resulted in an increase in the percentage of infected cells (Figure 5A,B). Additionally, untreated cells infected and then treated with SA for 30 min also demonstrated increased permissivity, if the addition of SA occurred within the first 4 h after infection (Figure 5A,B). However, the increase in permissivity was greatest if the SA was added prior to virus infection. Addition of SA at 6 h p.i. or later had no effect on the percentage of infected cells at 18 h p.i. (Figure 5A,B). These data suggest that SA is most beneficial when added early during the viral life cycle.

### 3.7. Preventing eIF2α De-Phosphorylation by Using Salubrinal has no Effect on Reovirus Infectivity

**Figure 6:**
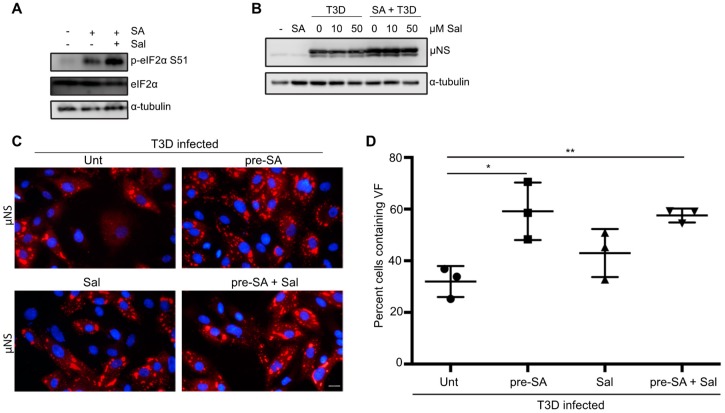
Salubrinal treatment does not negatively impact reovirus infection. (**A**) CV-1 cells were left untreated (-), or were incubated for 17.5 h in the presence or absence of 50 µM salubrinal (sal) before adding 0.5 mM sodium arsenite (SA) for 30 min. Following the removal of SA at 18 h p.i., the media was replaced and cells were incubated in the presence or absence of 50 µM sal for an additional 2 h. Cells were then lysed and the expression of the indicated proteins was determined by immunoblot. (**B**) CV-1 cells were either left untreated (-), were pre-treated with 0.5 mM SA for 30 min (SA), or were infected with T3D at an MOI = 10 in the presence or absence of SA pre-treatment. Immediately following infection, salubrinal was added to T3D-infected cells at the indicated concentrations. At 18 h p.i., cells were lysed and the expression levels of the indicated proteins was determined by immunoblot. (**C**) CV-1 cells were left untreated or were treated with 0.5 mM SA for 30 min prior to infection with T3D at an MOI = 1. Following infection, 50 µM sal was added where indicated. At 18 h p.i., cells were fixed and immunostained for μNS (red) and DAPI (nuclei, blue). (**D**) The percent of cells containing viral factories (VFs) as represented in panel (C) was quantified ((# of cells containing VFs/total # of cells) × 100) from three independent experiments. * *p* < 0.05; ** *p* < 0.01; two-tailed unpaired *t* test. The error bars indicate S.D. Scale bars, 20 μm.

Activation of HRI, PKR, PERK, or GCN2 kinases in response to stress results in the phosphorylation of eIF2α at serine position 51 and subsequent induction of the ISR. In response to the accumulation of p-eIF2α, part of the ISR includes expression of growth arrest and DNA damage protein-34 (GADD34). GADD34 is a protein phosphatase-interacting protein that, in conjunction with protein phosphatase 1 (PP1), acts to dephosphorylate eIF2α at serine 51 during times of cellular stress [34]. It was previously shown that GADD34 is upregulated in cells following infection with reovirus strains that induce host shut-off [15]. Our findings thus far are consistent with previous reports suggesting that eIF2α kinase activation is beneficial to reovirus infection [15]. We, therefore hypothesized that reovirus may be capable of viral protein synthesis in the face of enhanced eIF2α phosphorylation. We reasoned that inhibition of GADD34/PPI dephosphorylation would enhance phosphorylation of eIF2α, but should have no effect on viral protein synthesis. We used a selective inhibitor of GADD34, salubrinal, which prevents the activity of the GADD34/PPI complex without affecting the kinases that phosphorylate eIF2α [35]. To confirm that salubrinal inhibited eIF2α de-phosphorylation as expected, cells were either incubated in the presence or absence of 50 μM salubrinal for 17.5 h before treating with 0.5 mM SA for 30 min. Two hours following the removal of SA, cells were lysed and immunoblots were performed for the indicated proteins. As expected, treatment with SA resulted in an increase in eIF2α phosphorylation compared to untreated cells (Figure 6A). The expression level of p-eIF2α was enhanced even further when cells were incubated with salubrinal prior to SA treatment (Figure 6A). Next, we determined the impact of sustained eIF2α phosphorylation on reovirus protein expression. Immediately following adsorption of reovirus, CV-1 cells were treated with increasing amounts of salubrinal and cells were harvested at 18 h p.i. to determine viral protein expression (Figure 6B). Even at high concentrations, salubrinal had no impact on viral protein expression, suggesting that reovirus tolerates the cellular antiviral activity of eIF2α phosphorylation. Given that SA is a known inducer of eIF2α phosphorylation, we also tested the impact of adding salubrinal following reovirus adsorption to SA pre-treated cells (Figure 6B).

Again, we observed no negative impact on viral protein expression when cells were pre-treated with SA prior to infection and then incubated in the presence of salubrinal for the duration of infection (Figure 6B). Consistent with this, the percentage of infected cells was not impacted by the addition of 50 µM salubrinal to untreated cells or cells pre-treated with 0.5 mM SA (Figure 6C,D). Together, these data suggest that sustained eIF2α-phosphorylation resulting from salubrinal treatment during reovirus infection is not anti-viral. 

## 4. Discussion

In this report, we found that treatment of cells with SA, a potent inducer of the ISR and eIF2α phosphorylation, prior to inoculation with reovirus is beneficial to the virus in a strain-independent but cell-type-specific manner. SA treatment induces the formation of reactive oxygen species (ROS) within cells. The increased intracellular ROS in turn lead to the activation of HRI, which then phosphorylates eIF2α at serine residues 48 and 51, leading to general translation repression and downstream activation of the ISR. In order to mature and become activated, HRI has to be in a complex with CDC37, PPP5C, and HSP90. In addition to being activated by heme-deficiency, HRI is also activated by HS and osmotic shock, but not by ER stress or by amino acid or serum starvation [7]. In contrast to the enhanced viral replication we saw following pre-treatment with SA, HS pre-treatment did not enhance viral replication. HS also activates HRI, but to a qualitatively lower level than SA and with a different pattern of autophosphorylation of HRI [7]. Why pre-stressing cells with SA, but not with HS, benefited viral replication is unclear. It is possible that the kinetics, magnitude, and timing of eIF2α phosphorylation following these different stressors varied, leading to the different outcomes we saw. Alternatively, we cannot rule out the possibility that SA treatment activates responses not mediated by the ISR that benefit viral replication. 

In addition to inducing eIF2α phosphorylation, SA induces the formation of SGs. Consistent with the findings of Qin et al., we found that reovirus infection induced SG formation early after infection with the number of SG-containing cells reducing by 8 h and disappearing late in infection. Our data in CV-1 cells are not consistent with the report by Smith et al., who detected SGs at 19.5 h post-infection of DU145 cells [15,16]. While it is likely that different strains of reovirus induce SGs to varying extents, and that this is influenced by cell type and MOI, our data using the T3D strain suggests that infection with this strain of reovirus results in a low level of SG induction that is absent at late times post infection. The timing of SG appearance (peak at 6 h) and dissolution (beginning at 8 h) in our hands supports the idea put forth by Qin et al. that SG induction is linked to VF formation and that the onset of viral translation interferes with SG stability [16]. Smith et al. stained reovirus-infected cells at 19.5 h post-infection for the SG protein, TIAR, but did not co-stain for a viral protein, such as µNS, to detect VFs. We and others have observed that the SG protein, G3BP1, localizes to the outer margins of VFs at late times post infection in a fraction of reovirus infected cells [20]. Therefore, the TIAR-positive punctae seen at 19.5 h post infection by Smith may in fact have been VFs that were co-staining for the presence of TIAR [15]. The significance of SG protein relocalization to VFs remains to be determined. Still, these data imply that reovirus has evolved mechanisms to counter the cellular antiviral activity of translation suppression through SG induction. 

In our examination of SGs and SG proteins, we detected an increase in TIAR expression by immunoblot at 18 h p.i. compared to 2 h p.i. that was in contrast to both the immunofluorescence data and G3BP expression levels. Confounding this further, we observed an increase in TIAR expression in untreated cells harvested at 18 h compared to 2 h. Previous reports have indicated that during times of stress, SG protein levels remain constant but that their localization shifts [36,37,38]. TIAR is an RNA-binding protein that is found primarily in the nucleus of cycling cells and is involved in controlling mitotic entry but is capable of shuttling between the nucleus and the cytoplasm [39]. In response to stress, Fas-induced apoptosis, and transcriptional blockade, TIAR will accumulate in the cytoplasm [10,40,41]. Movement of TIAR from the nucleus has also been observed during herpes simplex virus 1 infection, with cytoplasmic accumulation beginning as early as 6 h after infection. In that study, a comparison of SG protein expression levels in the nucleus and cytoplasm revealed that although the cytosolic expression levels increased, total cellular protein levels were constant [42]. From this, we attribute the increase in TIAR expression at late times post infection to primarily be a result of the movement of TIAR into the cytoplasm. While we cannot rule out the possibility that the increased cytoplasmic expression is a result of viral infection, a similar pattern was detected in uninfected cells, suggesting this is more likely a result of transcriptional blockade associated with contact inhibition. 

Pre-treatment of cells with SA led to enhanced viral protein expression, percent infectivity, and viral titer. Given that reovirus compartmentalizes the translational machinery in VFs and that components of the translational machinery are also sequestered within SGs, we previously speculated that SGs may serve as a reservoir of translational machinery for reovirus VFs [19]. However, comparison of reovirus infection following pre-treatment with either SA or HS suggests that the benefit is independent of SG formation, as both treatments resulted in a robust induction of SGs, but only SA pre-treatment led to an increase in viral replication at late times p.i. Additionally, since we were unable to achieve robust SG formation following HS in CV-1 cells, viral replication studies were performed in L929 cells. Therefore, we cannot rule out cell-specific influences.

Our data is consistent with the findings of Smith et al. that reovirus benefits from activation of the ISR. In that study, the authors found that reovirus replicated to lower titers in cells lacking a phosphorylatable eIF2α [15]. SA is a potent activator of the ISR. SA-mediated activation of the HRI kinase leads to phosphorylation of eIF2α and downstream upregulation of ATF4 and other transcriptional mediators of the ISR [22]. Given that canonical cap-dependent cellular translation relies on the availability of active GTP.eIF2, it is possible that activation of the ISR allows reovirus mRNAs a competitive advantage for limited translational machinery. The effect of SA was greatest when added before reovirus adsorption but was still beneficial if treatment occurred within the first 4 h of infection. Translation of reovirus mRNAs rises sharply at ~ 6 h p.i. and peaks at ~12 h p.i. [33]. Given that the rising level of viral protein synthesis is concomitant with continued host protein synthesis, it is possible that SA treatment selectively reduces the translational machinery available for host protein synthesis [33]. It has been postulated that heightened eIF2α phosphorylation may more strongly affect mRNAs containing long or highly structured 5′ untranslated regions (UTRs) [31,43]. Reovirus mRNAs have short 5′ and 3′ UTRs, possibly providing protection to viral mRNAs during translation [44,45]. Our findings that reovirus protein expression and infectivity were unaffected when we treated cells with salubrinal to prevent de-phosphorylation of eIF2α further supports this and suggests that reovirus is refractory to salubrinal exposure. 

We cannot exclude the possibility that pre-treatment of cells with SA has other effects that promote viral replication independently of eIF2α phosphorylation. Reovirus is an oncolytic virus, preferentially infecting cancer cells. This phenomenon has been linked to a mutationally-active Ras pathway, however, reports have been conflicting regarding Ras dependency [46,47,48,49,50]. Cancer cells have elevated levels of chaperones that facilitate the high levels of protein synthesis typical of transformed cells. The efficiency of mRNA translation is increased in the presence of supplemental recombinant Hsc70 and the rate of translation elongation may be regulated by chaperone availability [51]. Treatment with SA also increases Hsc70/Hsp70 chaperone levels in cells [52]. Furthermore, Hsc70 is required for HRI activation and blockade of Hsc70 disrupts HRI activation [7]. We have previously reported that Hsc70 is specifically targeted to the VF, the site of reovirus translation, although why this protein is specifically recruited remains unclear as its recruitment is independent of its chaperone function [19,53]. Alternatively, SA pre-treatment might alter the availability of Hsc70 or other protein folding chaperones in a way that is favorable during the early stages of reovirus replication. Hsc70 was previously shown to promote release of the δ fragment from the reovirus outer capsid protein μ1, completing disassembly of the outer capsid and deposition of a transcriptionally active core particle in the cytosol [54]. In that study, δ fragment release was nearly abolished in rabbit reticulocyte lysates immunodepleted of Hsc70 and Hsp70, but could be restored by the addition of purified Hsc70, suggesting a role for Hsc70 in virus uncoating [54].

Infection with reovirus results in increased expression of stress response genes, including *Hsc70*, and *GADD34*, the latter of which complexes with PP1 to reverse eIF2α phosphorylation. We observed that type 3 Dearing (T3D) reovirus protein expression was unaffected in the presence of salubrinal, a selective inhibitor of the GADD34/PP1α complex responsible for dephosphorylating eIF2α, even in cells pre-treated with SA to elevate the level of phosphorylated eIF2α. The observation that salubrinal was not anti-viral to T3D reovirus is not unique. Rotavirus, a fellow member of the Reoviridae family, as well as other viruses, including hepatitis C virus and mouse hepatitis coronavirus, demonstrate continued viral protein synthesis despite heightened eIF2α phosphorylation [55,56,57]. While this provides additional evidence that reovirus benefits from the ISR, it is important to note that reovirus-induced expression of stress response genes is not uniform and that host shutoff strains, including C8 and C87 (type 3 Abney, T3A), induce increased expression of these genes compared to T3D, which is not considered a host shutoff strain. While all reovirus strains tested, including T3A, benefited from SA pre-treatment, host shutoff strains may respond differently from T3D to salubrinal treatment [15].

In addition to strain-specific differences in the ability to induce host shutoff, cell differences have also been recorded. For instance, reovirus-induced host shutoff was not observed in HeLa cells but was in L929 cells [33]. This may be linked to our observation that T3D infection in HeLa cells was unaffected by SA pre-treatment, whereas SA pre-treatment was beneficial in other cell types including L929 cells, CV-1, and HPDE cells.

Overall, this study finds that SA-induced activation of the ISR prior to reovirus inoculation results in enhanced viral protein expression, percent infectivity, and viral yield. Furthermore, activation of the ISR is insufficient for replication enhancement as both HS and SA activate the ISR, but only SA treatment was associated with increased viral infectivity and higher yield. Finally, SA-induced enhancement was observed across reovirus strains but was not observed in all cell types, suggesting a role for cell type-specific influences. Understanding the interplay between reovirus and the ISR, and how to modulate it to enhance virus replication, could reveal novel targets to strengthen the oncolytic potential of reovirus.

## Supplementary Materials

The following are available online at https://www.mdpi.com/1999-4915/11/6/563/s1, Figure S1: Reovirus infection induces the formation of stress granules (SGs) at early times post infection (A) CV-1 cells were mock infected, pre-treated with 0.5 mM sodium arsenite (SA) for 30 min, or infected with T3D at an MOI = 10. At the indicated times, cells were fixed and co-immunostained for G3BP (red) and TIAR (green), upper panels, to detect SGs in uninfected cells or TIAR (green) and μNS (red), lower panels, to detect SGs in T3D-infected cells. Cell nuclei were stained with DAPI (blue). Mock and SA-treated cells were fixed at 18 h. Scale bars, 20 μm. Figure S2: Induction of the ISR (A) CV-1, HeLa, L929, or HPDE cells were either left untreated or were pre-treated with 0.5 mM sodium arsenite (SA) for 30 min. Following this, cells were fixed and co-immunostained for G3BP (red) and TIAR (green) to detect SGs. Cell nuclei were stained with DAPI (blue). Scale bars, 10 μm. (B) CV-1, HeLa, or L929 cells were either left untreated, treated with SA as in (A), or heat shocked at 44 °C for 45 min. Following stress treatments, samples were harvested and lysates were immediately combined with SDS-PAGE loading buffer. Proteins were resolved by SDS-PAGE and immunoblots were performed to determine the expression level of indicated proteins.
