# Supplementary material for: Mammalian orthoreovirus Infection is Enhanced in Cells Pre-Treated with Sodium Arsenite
Source: Viruses. 2019 Jun 18;11(6):563. doi: 10.3390/v11060563 (PMC6631071; doi:10.3390/v11060563)
Supplement: Supplementary file 1 [file viruses-11-00563-s001.pdf]

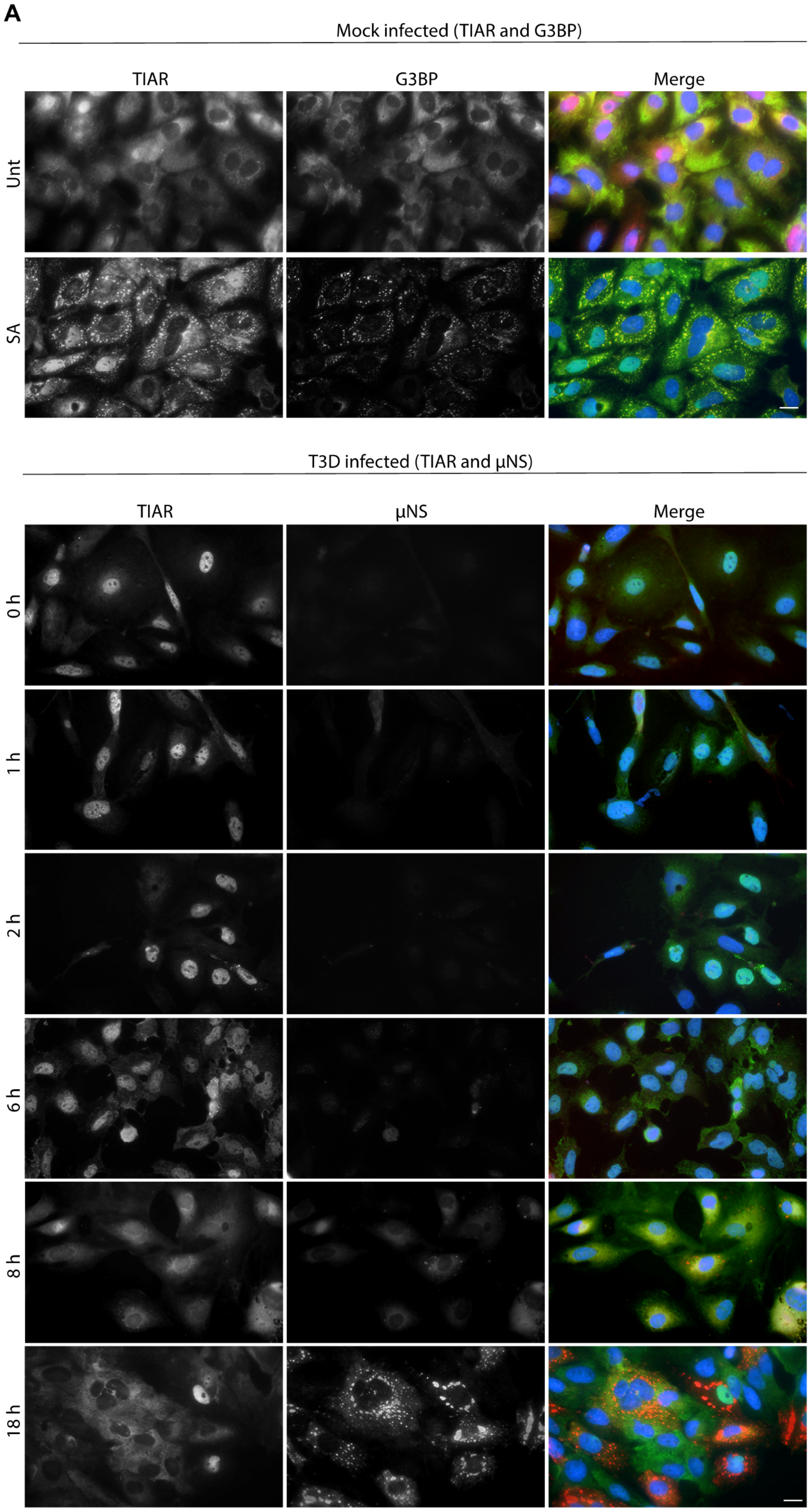
A
Mock infected (TIAR and G3BP)
TIAR
G3BP
Merge
Unt
SA
T3D infected (TIAR and μNS)
TIAR
μNS
Merge
0 h
1 h
2 h
6 h
8 h
18 h

Figure S1: Reovirus infection induces the formation of stress granules (SGs) at early times post infection. (**A**) CV-1 cells were mock infected, pre-treated with 0.5 mM sodium arsenite (SA) for 30 min, or infected with T3D at an MOI = 10. At the indicated times, cells were fixed and co-immunostained for G3BP (green) and TIAR (red), upper panels, to detect SGs in uninfected cells or TIAR (green) and μNS (red), lower panels, to detect SGs in T3D-infected cells. Cell nuclei were stained with DAPI (blue). Mock and SA-treated cells were fixed at 18 h. Scale bars, 20 μm.

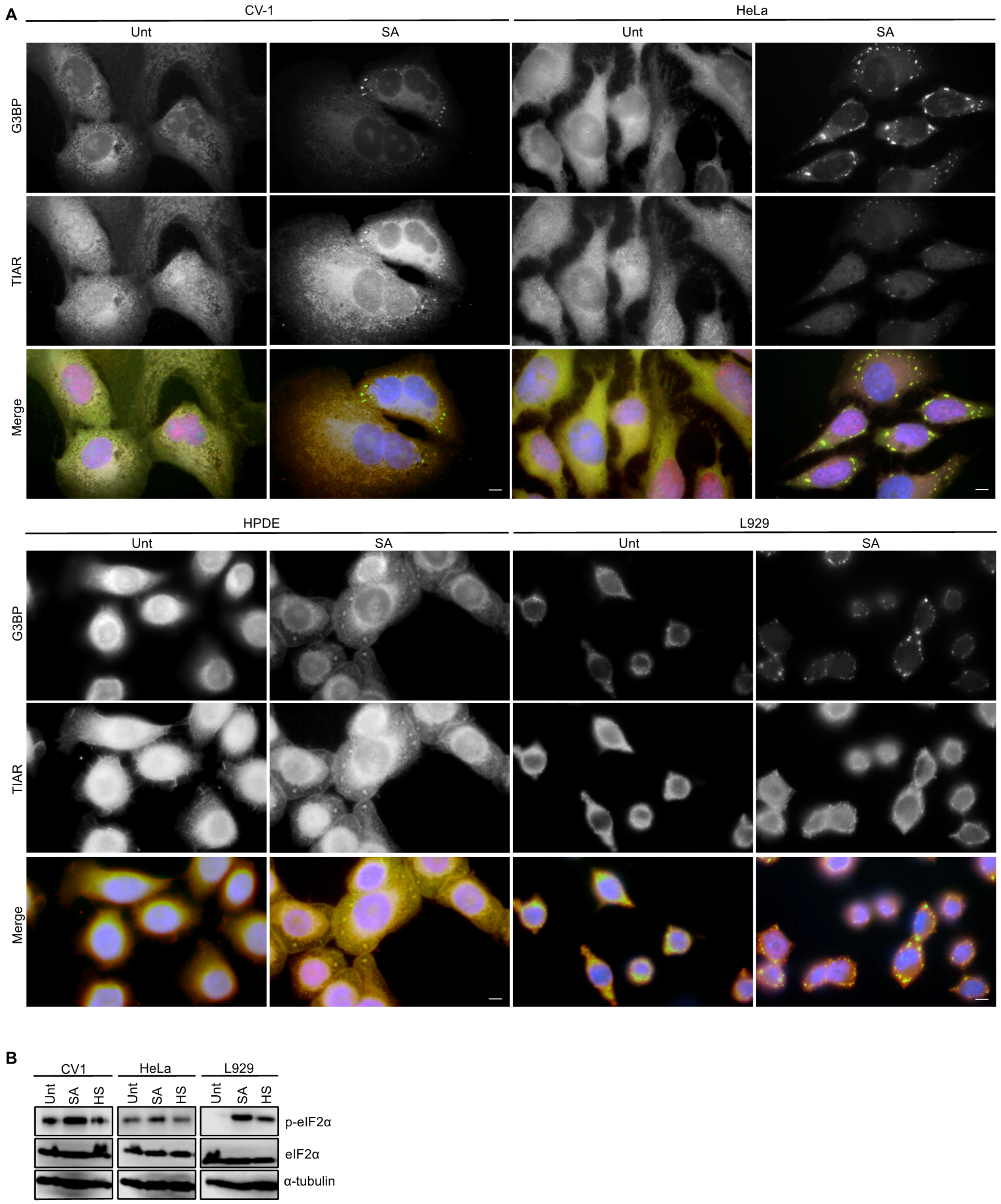

Figure S2: Induction of the ISR. (**A**) CV-1, HeLa, L929 or HPDE cells were either left untreated or were pre-treated with 0.5 mM sodium arsenite (SA) for 30 min. Following this, cells were fixed and co-immunostained for G3BP (green) and TIAR (red) to detect SGs. Cell nuclei were stained with DAPI (blue). (**B**) CV-1, HeLa or L929 cells were either left untreated, treated with SA as in (A) or heat shocked at 44°C for 45 min. All treatments were performed such that all samples were harvested collectively. Immediately following lysis, proteins were resolved by

SDS-PAGE and immunoblots were performed to determine the expression level of indicated proteins. Scale bars, 10 μm.
